# Important gene–gene interaction of *TNF-α* and *VDR* on osteoporosis in community-dwelling elders

**DOI:** 10.1371/journal.pone.0226973

**Published:** 2019-12-30

**Authors:** Li-Na Liao, Chia-Ing Li, Fang-Yang Wu, Chuan-Wei Yang, Chih-Hsueh Lin, Chiu-Shong Liu, Wen-Yuan Lin, Tsai-Chung Li, Cheng-Chieh Lin

**Affiliations:** 1 Department of Public Health, College of Public Health, China Medical University, Taichung, Taiwan; 2 Department of Medical Research, China Medical University Hospital, Taichung, Taiwan; 3 School of Medicine, College of Medicine, China Medical University, Taichung, Taiwan; 4 Department of Family Medicine, China Medical University Hospital, Taichung, Taiwan; 5 Department of Healthcare Administration, College of Medical and Health Sciences, Asia University, Taichung, Taiwan; GeneDx, UNITED STATES

## Abstract

Gene effects on osteoporosis have been studied separately and may have been masked by gene–gene and gene–environment interactions. We evaluated gene–gene and gene–physical activity interactions of the variants of tumor necrosis factor-α (*TNF-α*) and vitamin D receptor (*VDR*) genes on osteoporosis. A total of 472 elders were included. Seven variants (*TNF-α*: rs1799964, rs1800629, rs3093662; *VDR*: rs7975232, rs1544410, rs2239185, rs3782905) were genotyped. Bone mineral densities of the lumbar spine, femoral neck, and total hip were measured by dual-energy X-ray absorptiometry. Predictive models’ ability to discriminate osteoporosis status was evaluated by areas under the receiver operating characteristics (AUROC) curve. After multivariable adjustment, significant interactions of *TNF-α* rs1800629 and *VDR* rs3782905 were observed on overall and lumbar spine osteoporosis. In elderly women, we found that those carrying the CG/CC genotype of *VDR* rs3782905 were significantly associated with increased odds of overall osteoporosis compared with those carrying the GG genotype of *VDR* rs3782905 among those carrying *TNF-α* rs1800629 GG genotype. The adjusted odds ratios (ORs) for *VDR* rs3782905 CG/CC genotype in elderly women carrying *TNF-α* rs1800629 AG/AA and GG genotypes were 0.1 (0.01, 0.98) and 3.54 (1.51, 8.30), respectively. We observed significant differences in AUROCs between the model with traditional covariates plus variants and their interaction term and the model with traditional covariates only (AUROCs: 0.77 and 0.81; *p* = 0.028). Although the sample size of this study may have been relatively small, our results suggest that the interaction of the CG/CC genotype of *VDR* rs3782905 with *TNF-α* rs1800629 GG genotype was associated with increased odds of overall and lumbar spine osteoporosis in elderly women.

## Introduction

Cytokine activation during inflammation plays an important role in osteoporosis-related aging processes in the elderly [1]. Quantitative and qualitative age-related bone changes take place during normal aging. Aging is linked to chronically elevated tumor necrosis factor (TNF)-α level among proinflammatory cytokines [2]. TNF-α is an important bone metabolism regulator [3, 4]. Increased biomarkers of inflammation, such as TNF- α, which initiates the inflammatory cascade, are negatively related to bone mineral density (BMD) through aging and estrogen [5–8]. TNF-α is a pleiotropic cytokine that is fundamental for bone remodeling [9, 10] and increases bone loss by directly activating osteoclast precursors or by inducing the receptor–activator production of NF-κB ligand by osteoblasts [11].

Genetic variations play a crucial role in inflammation and may alter immune response intensity and affect the susceptibility of age-related diseases by regulating gene transcription or reducing the production of proinflammatory cytokines [12, 13]. Genetic variants in the *TNF-α* promoter region are related to TNF-α serum levels; these variants include single nucleotide polymorphisms (SNPs) rs1799964 (-1031C/G) and rs1800629 (-308G/A). rs3093662 in the first intron found to be associated with TNF-α production [14]. Prior studies found that the SNPs in *TNF-α* gene are related to osteoporosis in elderly people [15–20]. Moreover, the allelic variants of the *TNF-α* gene and BMD, including those between SNPs rs1800630 (-863C/A) and rs1800630 (-1031T/C) of the *TNF-α* gene and low BMD [16, 17] are associated to a dinucleotide repeat polymorphism in *TNF-α* for linkage to osteoporosis [21]. The *TNF-α* gene may have joint and interactive effects with LEPR in osteoporosis development [11, 20].

Vitamin D has anti-inflammatory properties and might influence the regulation of immune functions and the proliferation and differentiation of many cell types [22]. Vitamin D suppresses TNF-α-induced inflammation through vitamin D receptor (VDR) in animal studies [23]. A prior animal study revealed that the effects of vitamin D resulted in suppressing inflammation-related gene expression, including a decrease in TNF-α gene expression that was induced by high-intensity exercise [24] *In vitro* studies showed that calcitriol, the hormonally active metabolite of vitamin D, can inhibit the release of TNF- α in a dose-dependent manner [25]. The *VDR* gene is located in the chromosome 12q13 region [26]. Furthermore, the VDR receptor is expressed in human lungs throughout the full epithelial layer and main receptor to form the active vitamin D metabolite, 1α,25-dihydroxyvitamin D_3_ (1α,25[OH]_2_D_3_) [27]. *VDR* variants are associated with BMD or low BMD [28–33]. A recent meta-analysis pooled the results on the associations between *VDR* gene variants and osteoporosis and BMD in postmenopausal women. The findings indicated that *VDR* ApaI is linked with a decreased risk of osteoporosis in Caucasian postmenopausal women. By contrast, Asian postmenopausal women with *VDR* BsmI and *VDR* FokI tend to have an increased risk of osteoporosis [28]. In our previous work, we reported that *VDR* variants and physical activity have a joint effect on osteoporosis in Chinese elders. Mencej–Bedrac reported the individual and interactive effects of variants in the *VDR* gene, *OPG*, and *TNFSF11* on BMD stratified by gender and osteoporosis status [34]. However, no prior study has explored the independent and interactive effect of the genetic variation of *VDR* and *TNF-α* genes on BMD and osteoporosis. The present study aims to evaluate the independent and interactive effects of SNPs in *VDR* and *TNF-α* genes on BMD and osteoporosis in elders from the Taichung Community Health Study for Elders (TCHS-E) in Taichung City, Taiwan.

## Materials and methods

### Study subjects

A community-based cross-sectional study was conducted among elders from TCHS-E, who aged 65 years and older and are residing in eight LIs (administrative neighborhoods) of Taichung City in June 2009. A total of 3,997 elders resided in these LIs during the study period and accounted for 4.58% of citizens of the same age group in Taichung. The Bureau of Households provided the list of all the records of potential, eligible study subjects for the sampling frame. We invited all eligible elders to participate and excluded 1,764 ineligible elders for the following reasons: dying or being hospitalized, residing in a nursing home, and moving out of the study area. Finally, 855 elders aged 65–98 completed a physical check-up and answered a questionnaire. We further excluded elders with missing data on height, weight, and BMD, thus resulting in a final number of 844 elders. A total of 480 participating, unrelated elders provided DNA specimens for genotyping, in which 472 were successful. The detailed methodology of this procedure was reported in a separate study [35]. This study was conducted after obtaining approval from the Institutional Review Board of China Medical University Hospital (DMR97-IRB-055) and all methods were performed in accordance with the relevant guidelines and regulations. Each participant provided his/her written informed consent.

### Bone mineral density and osteoporosis

BMD was measured by dual energy X-ray absorptiometry (DXA) system (GE-LUNAR DPX, Lunar Corporation, Madison, WI) at the left and right total hip, left and right femoral neck, anteroposterior lumbar spine (L1–L4), Ward’s triangle, and trochanter of central skeletal sites. Lunar enCORE2004 software version 8.60.006 was used to analyze the physical phantom data for DXA machine calibration every day. The coefficient variation of all diverse sites was <1%. Each subject was asked to lie down in a supine position in their underwear with no metal items to obtain a whole-body scan. The whole-body composition analysis comprised data on various regions, such as the spine, neck, arms, legs, and torso. The value for the total hip and femoral neck measurement was calculated by the lowest value of BMD between the right- and left-side values of each participant’s BMD. The lowest value of BMD among lunar lumbar spine, femoral neck, and total hip sites was defined as his/her overall BMD value. The WHO diagnostic criterion of BMD less than 2.5 SDs below young adult mean (-2.5 T-score) was used to define osteoporosis [36].

### Measurements

#### SNP selection and genotyping

We genotyped seven tag-SNPs, consisting of four *VDR* SNPs [rs7975232 (*Apa*I), rs3782905 (*Dde*l), 2239185, and rs1544410 (*Bsm*I) in the intron of *VDR* gene] and three *TNP-α* SNPs (rs1800629, rs3093662, and rs1799964) according to previous studies for the Han population in Beijing, China in the international HapMap Project. Genomic DNA was extracted from the blood samples by using a commercial kit (QIAamp DNA Blood kit; Qiagen, Chatsworth, CA, USA). An ND-2000c spectrophotometer (NanoDrop Technologies, Wilmington, DE, USA) quantified the concentration of the purified DNA. All tag-SNPs were genotyped by the GoldenGate assay of Illumina Inc. (San Diego, CA, USA). The quality of genomic DNA specimen was evaluated in advance to improve the genotyping success rate.

#### Covariates

Data on sociodemographic factors and lifestyle behaviors were collected through self-administered questionnaires. Physical activity for leisure time was measured by a battery of 34 items for different kinds of physical activities performed by an elder. We asked about the average time spent on each type of activity on a weekly basis and about all the activities performed during the past week. We classified elders as physically active if they currently perform a regular leisure activity at least once a week for at least 30 minutes in the last 6 months. The height and weight of a subject in bare feet were measured by an autoanthropometer (superview, HW-666). BMI was derived by dividing the subject’s weight (kg) by the square of the height (m).

### Statistical analysis

Descriptive statistics of mean, standard deviation, and proportion were used in this study, and bivariate analyses of Chi-squared and t-tests were used to compare the characteristics among elders with osteoporosis at various sites. Hardy-Weinberg equilibrium (HWE) law was first evaluated by an exact Chi-squared test for goodness of fit, stratified by gender for each tag-SNP by a PLINK software (v1.07, http://pngu.mgh.harvard.edu/purcell/plink). We then examined the main effects of genotype for seven SNPs on BMD T-score and serum TNF-α level via ANOVA. Next, we explored the gene–gene interactions on osteoporosis. For each SNP, the major–major and the minor–major/minor–minor genotypes were treated as reference and indicator, respectively. The product term of two SNPs, i.e., one from *TNP-α* gene and one from *VDR* gene, was derived to assess the interaction between two SNPs, with the main effect of these two SNPs being simultaneously entered into the multivariate logistic regression analysis that considered age, physical activity, BMI, and smoking and drinking habits. Odds ratios (ORs) and 95% confidence intervals (CIs) were presented. Finally stratified analysis was performed for significant interaction. The p-values corrected for false discovery rate method were reported to consider multiple-testing problems. Area under the receiver operating characteristic (AUROC) curve was used to assess predictive accuracy and the discriminatory ability of prediction models with and without considering gene–gene and gene–physical activity interactions. Significance level was set at a two-tailed *p* < 0.05. All analyses were performed using gender stratification via Statistical Analysis System software (v9.4, SAS Institute Inc., Cary, NC, USA).

## Results

The characteristics of study subjects according to osteoporosis status at various sites stratified by gender are presented in Table 1. A total of 110 elderly persons with overall osteoporosis (36 men and 67 women) and 369 elderly persons without overall osteoporosis (215 men and 154 women) were involved in this study. Elderly men or women with osteoporosis at various sites were generally older and had lower BMI values, BMD T-scores, and proportions of having regular physical compared with those who do not have osteoporosis.

**Table 1:** Characteristics of study subjects according to osteoporosis status at various sites stratified by gender.

Characteristic	Overall		Lumbar spine		Femoral neck		Total hip	
	OST cases	Controls	OST cases	Controls	OST cases	Controls	OST cases	Controls
**Women (N = 221)** N (%)	67 (30.3)	154 (69.7)	52 (23.6)	168 (76.4)	32 (14.6)	187 (85.4)	27 (12.4)	191 (87.6)
Age (years)	75.3 ± 6.1*	71.7 ± 4.7*	75.1 ± 6.4*	72.1 ± 5.0*	76.0 ± 6.4*	72.1 ± 4.8*	76.9 ± 5.3*	72.0 ± 5.0*
BMI (kg/m^2^)	21.7 ± 3.2*	24.3 ± 3.1*	21.7 ± 3.2*	24.0 ± 3.2*	21.6 ± 3.5*	23.8 ± 3.2*	20.7 ± 3.2*	23.9 ± 3.2*
TNF-α (pg/mL)	3.2 ± 4.8	3.5 ± 8.1	3.3 ± 5.3	3.4 ± 7.7	2.9 ± 3.1	3.5 ± 7.8	4.5 ± 6.8	3.3 ± 7.4
BMD T-score	-3.1 ± 0.5*	-1.3 ± 0.8*	-3.2 ± 0.4*	-0.8 ± 1.1*	-2.9 ± 0.5*	-1.2 ± 0.9*	-2.9 ± 0.6*	-0.9 ± 1.0*
**Behaviors**								
Physical activity	43 (64.2)*	124 (80.5)*	35 (67.3)	131 (78.0)	18 (56.3)*	149 (79.7)*	13 (48.2)*	154 (80.6)*
Smoking	2 (3.0)	5 (3.3)	0 (0.0)	7 (4.2)	2 (6.3)	5 (2.7)	0 (0.0)	7 (3.7)
Alcohol drinking	4 (6.0)	10 (6.5)	4 (7.7)	9 (5.4)	1 (3.1)	13 (7.0)	1 (3.7)	13 (6.8)
**Men (N = 251)** N (%)	36 (14.3)	215 (85.7)	25 (10.0)	225 (90.0)	21 (8.4)	229 (91.6)	12 (4.8)	237 (95.2)
Age (years)	76.7 ± 7.3*	74.4 ± 6.3*	75.6 ± 6.4	74.5 ± 6.3	79.1 ± 7.5*	74.3 ± 6.2*	77.2 ± 4.6	74.6 ± 6.5
BMI (kg/m^2^)	20.6 ± 3.2*	24.0 ± 2.9*	20.8 ± 3.1*	23.9 ± 3.0*	19.9 ± 3.0*	23.9 ± 3.0*	19.0 ± 2.3*	23.8 ± 3.1*
TNF-α (pg/mL)	4.3 ± 6.9	5.2 ± 15.4	4.1 ± 6.7	5.1 ± 15.1	3.7 ± 7.4	5.1 ± 14.9	6.1 ± 11.0	4.8 ± 14.6
BMD T-score	-2.9 ± 0.4*	-1.0 ± 0.9*	-2.9 ± 0.4*	-0.1 ± 1.6*	-2.9 ± 0.3*	-0.9 ± 0.9*	-2.7 ± 0.1*	-0.3 ± 1.1*
**Behaviors**								
Physical activity	25 (69.4)*	184 (85.6)*	16 (64.0)*	192 (85.3)*	14 (66.7)*	194 (84.7)*	9 (75.0)	198 (83.5)
Smoking	17 (47.2)	76 (35.4)	10 (40.0)	82 (36.4)	10 (47.6)	82 (35.8)	6 (50.0)	85 (35.9)
Alcohol drinking	12 (33.3)	68 (31.6)	7 (28.0)	73 (32.4)	6 (28.6)	73 (31.9)	6 (50.0)	73 (30.8)

Data were presented as mean ± SD for continuous variables or n (%) for categorical variables.

BMI: body mass index; TNF-α: tumor necrosis factor α; BMD: bone mineral density; OST: osteoporosis.

*p < 0.05 between cases and controls; two-sample t-test for continuous variables or chi-square test for categorical variables.

The two minor-allele frequencies (MAFs) in the *TNF-α* gene and four SNPs in the *VDR* gene were greater than 5.0% in our sample. The genotype frequencies of three *TNF-α* SNPs and three *VDR* SNPs in men and women followed the HWE law (all SNPs *p* > .05). The serum TNF-α level of men carrying the AC genotype of *VDR* rs7975232 was significantly lower than those of the AA genotype (P<0.05). We did not observe significant difference in BMD T-score at various sites (Table 2).

**Table 2:** Genotype and allele distributions of study subjects as well as serum TNF-α level and bone mineral density T-score distributions according to genotype status stratified by sex^a^.

Genotype or allele	n (%)	TNF-α (pg/mL)	BMD T-score			
			Overall	Lumbar spine	Femoral neck	Total hip
**Women**						
*TNF-α* rs1799964						
AA	139 (62.9)	3.13 ± 5.67	-1.72 ± 1.14	-1.25 ± 1.42	-1.38 ± 1.06	-1.13 ± 1.18
GA	73 (33)	4.05 ± 9.84	-2.07 ± 1.03	-1.62 ± 1.34	-1.60 ± 0.97	-1.36 ± 1.00
GG	9 (4.1)	2.66 ± 3.25	-1.42 ± 1.18	-1.28 ± 1.17	-0.92 ± 1.19	-0.67 ± 1.10
G*	91 (20.6)					
*TNF-α* rs1800629						
GG	167 (77)	3.58 ± 7.87	-1.86 ± 1.04	-1.38 ± 1.34	-1.49 ± 0.95	-1.22 ± 1.05
AG	48 (22.1)	2.98 ± 5.30	-1.72 ± 1.33	-1.38 ± 1.58	-1.23 ± 1.28	-1.09 ± 1.36
AA	2 (0.9)	2.86 ± 0.57	-1.60 ± 1.27	-0.90 ± 2.26	-1.35 ± 0.92	-0.60 ± 0.14
A*	52 (12)					
*TNF-α* rs3093662						
AA	212 (95.9)	3.52 ± 7.41	-1.82 ± 1.13	-1.36 ± 1.41	-1.42 ± 1.05	-1.17 ± 1.14
GA	9 (4.1)	1.19 ± 0.88	-2.07 ± 0.76	-1.76 ± 0.90	-1.78 ± 0.77	-1.53 ± 0.74
GG	0 (0.0)	–	–	–	–	–
G*	9 (2.0)					
*VDR* rs7975232						
CC	106 (50.5)	3.04 ± 5.84	-1.80 ± 1.10	-1.43 ± 1.35	-1.40 ± 0.95	-1.15 ± 1.06
AC	81 (38.6)	2.72 ± 4.73	-1.89 ± 1.19	-1.42 ± 1.41	-1.49 ± 1.22	-1.25 ± 1.27
AA	23 (11.0)	6.31 ± 15.74	-1.85 ± 0.87	-1.44 ± 1.12	-1.38 ± 0.80	-1.13 ± 0.92
A*	127 (30.2)					
*VDR* rs1544410						
GG	189 (86.7)	3.62 ± 7.77	-1.81 ± 1.09	-1.33 ± 1.39	-1.46 ± 1.00	-1.19 ± 1.10
AG	28 (12.8)	2.33 ± 2.90	-1.86 ± 1.26	-1.57 ± 1.45	-1.20 ± 1.31	-1.07 ± 1.35
AA	1 (0.5)	2.73	-3.40	-2.60	-3.40	-2.40
A*	30 (6.9)					
*VDR* rs2239185						
GG	111 (50.5)	3.07 ± 5.74	-1.82 ± 1.14	-1.45 ± 1.38	-1.35 ± 1.04	-1.11 ± 1.12
AG	93 (42.3)	4.14 ± 9.23	-1.85 ± 1.13	-1.28 ± 1.46	-1.59 ± 1.06	-1.33 ± 1.16
AA	16 (7.3)	1.84 ± 2.22	-1.76 ± 0.92	-1.46 ± 1.03	-1.16 ± 0.90	-0.92 ± 0.89
A*	125 (28.4)					
*VDR* rs3782905						
GG	149 (67.4)	3.11 ± 5.93	-1.74 ± 1.05	-1.31 ± 1.34	-1.36 ± 0.99	-1.10 ± 1.05
CG	68 (30.8)	4.17 ± 9.72	-1.96 ± 1.23	-1.46 ± 1.47	-1.59 ± 1.17	-1.33 ± 1.27
CC	4 (1.8)	2.33 ± 2.49	-2.75 ± 1.05	-2.45 ± 1.65	-1.70 ± 0.65	-1.80 ± 0.81
C*	76 (17.2)					
**Men**						
*TNF-α* rs1799964						
AA	155 (62.0)	5.70 ± 17.88	-1.27 ± 1.10	-0.39 ± 1.77	-1.03 ± 1.07	-0.35 ± 1.20
GA	87 (34.8)	3.88 ± 5.45	-1.35 ± 1.11	-0.44 ± 1.58	-1.10 ± 1.04	-0.40 ± 1.16
GG	8 (3.2)	5.82 ± 11.08	-1.38 ± 0.95	-0.29 ± 1.92	-1.33 ± 0.88	-0.80 ± 1.36
G*	103 (20.6)					
*TNF-α* rs1800629						
GG	206 (83.7)	5.16 ± 15.63	-1.30 ± 1.05	-0.40 ± 1.64	-1.09 ± 1.00	-0.42 ± 1.11
AG	40 (16.3)	4.60 ± 7.87	-1.19 ± 1.29	-0.33 ± 2.02	-0.85 ± 1.25	-0.11 ± 1.48
AA	0 (0.0)	–	–	–	–	–
A*	40 (8.1)					
*TNF-α* rs3093662						
AA	241 (96.0)	5.13 ± 14.76	-1.31 ± 1.11	-0.45 ± 1.69	-1.06 ± 1.07	-0.38 ± 1.20
GA	10 (4.0)	3.13 ± 4.30	-1.13 ± 0.69	0.31 ± 2.05	-1.05 ± 0.60	-0.50 ± 0.92
GG	0 (0.0)	–	–	–	–	–
G*	10 (2.0)					
*VDR* rs7975232						
CC	119 (49.2)	4.26 ± 6.59	-1.23 ± 1.09	-0.20 ± 1.80	-0.98 ± 1.04	-0.28 ± 1.14
AC	93 (38.4)	4.98 ± 6.67^b^	-1.38 ± 1.14	-0.67 ± 1.59	-1.14 ± 1.07	-0.47 ± 1.25
AA	30 (12.4)	9.25 ± 37.83	-1.29 ± 1.06	-0.32 ± 1.65	-1.05 ± 1.10	-0.43 ± 1.17
A*	153 (31.6)					
*VDR* rs1544410						
GG	220 (88.7)	5.06 ± 15.23	-1.29 ± 1.11	-0.39 ± 1.71	-1.05 ± 1.05	-0.35 ± 1.19
AG	27 (10.9)	4.49 ± 8.02	-1.50 ± 1.06	-0.66 ± 1.74	-1.23 ± 1.15	-0.67 ± 1.20
AA	1 (0.4)	5.56	-1.40	-0.60	-1.40	-0.90
A*	29 (5.8)					
*VDR* rs2239185						
GG	120 (48.0)	4.26 ± 6.58	-1.25 ± 1.09	-0.21 ± 1.80	-1.00 ± 1.04	-0.30 ± 1.15
AG	99 (39.6)	4.79 ± 6.96	-1.36 ± 1.14	-0.66 ± 1.62	-1.11 ± 1.09	-0.43 ± 1.26
AA	31 (12.4)	8.97 ± 36.98	-1.37 ± 1.01	-0.42 ± 1.58	-1.21 ± 1.01	-0.60 ± 1.11
A*	161 (32.2)					
*VDR* rs3782905						
GG	186 (74.4)	5.50 ± 16.40	-1.32 ± 1.06	-0.46 ± 1.61	-1.05 ± 1.03	-0.40 ± 1.14
CG	57 (22.8)	3.59 ± 5.65	-1.24 ± 1.18	-0.31 ± 1.86	-1.10 ± 1.12	-0.28 ± 1.30
CC	7 (2.8)	5.62 ± 12.13	-1.60 ± 1.27	-0.39 ± 2.86	-1.47 ± 1.18	-0.87 ± 1.51
C*	71 (14.2)					

TNF-α: tumor necrosis factor α; BMD: bone mineral density.

*: Minor allele.

^a^: All p > .05 from Hardy-Weinberg Equilibrium test.

^b^: p < .05 for comparing with AA genotype using Bonferroni multiple comparison tests.

**Fig 1:**
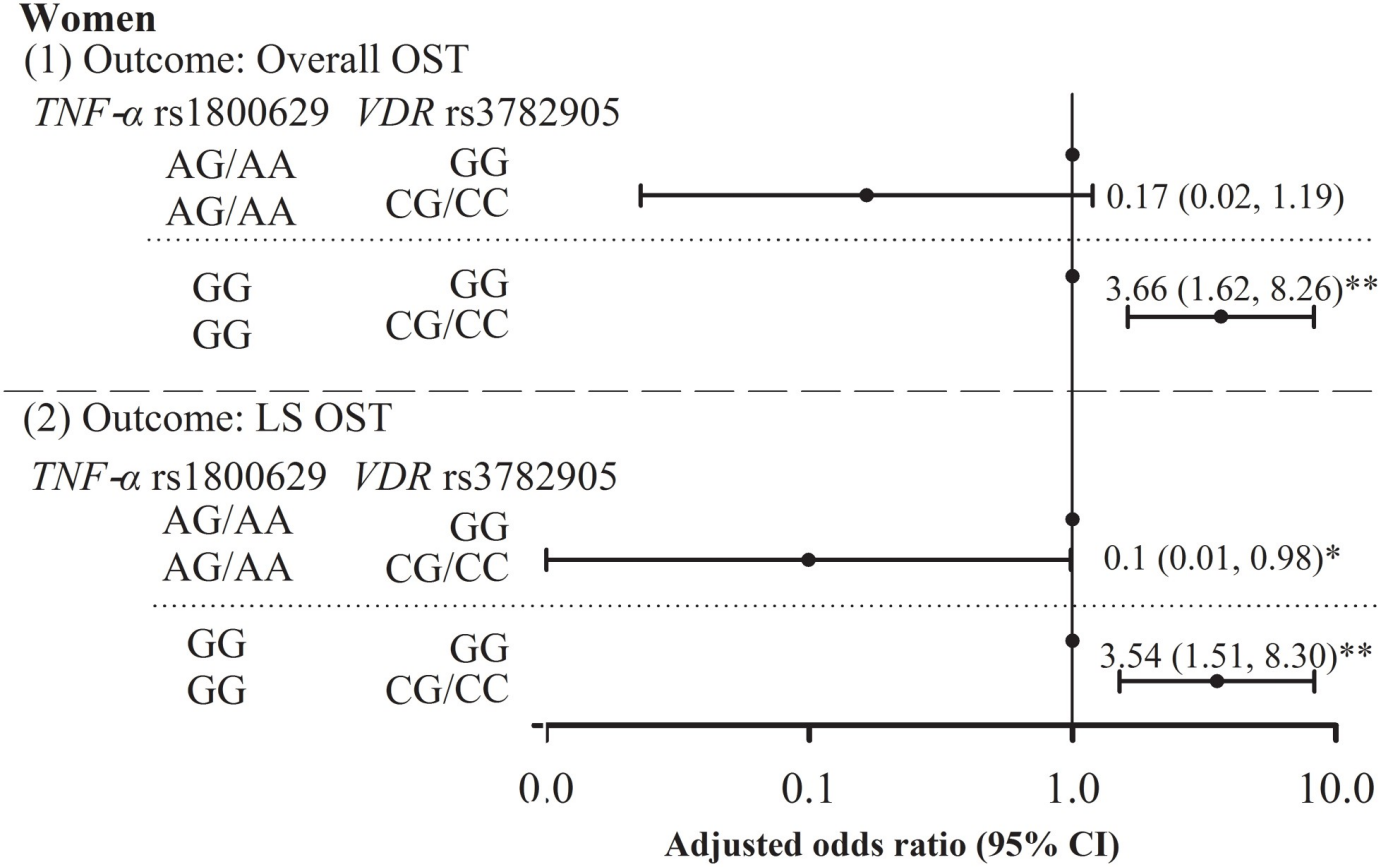
Stratified analysis by *TNF-α* rs1800629 for significant interaction of *VDR* rs3782905 with *TNF-α* rs1800629 on overall OST (1) and LS OST (2) among women is shown. The adjusted ORs were obtained from the model with consideration of SNP, age, BMI, physical activity, smoking, and drinking. *p < 0.05. OST: osteoporosis; LS: lumbar spine.

Table 3 presents the bi-locus interactions of *TNF-α* SNPs with *VDR* SNPs for exploring biological interactions. We detected significant interactions between *TNF-α* SNP rs1800629 and *VDR* rs3782905 on overall and lumbar spine osteoporosis in women after covariate adjustment and type 1 error correction (all *p* < 0.05). We further explored these significant interactions by estimating the adjusted OR in osteoporosis according to *TNF-α* status for *VDR* genotype (minor–major/minor–minor and major–major genotypes) (Fig 1). We found that the odds of overall osteoporosis among elderly women carrying CG/CC genotype of *VDR* rs3782905 were significantly higher than those among carrying *TNF-α* rs1800629 GG genotype (adjusted OR: 3.66 [95% CI: 1.62–8.26] but not among those carrying *TNF-α* rs1800629 AG/AA genotype. For lumbar spine, the effects of *VDR* rs3782905 were protective among elderly women carrying *TNF-α* rs1800629 AG/AA genotype, whereas its effectiveness was associated with increased odds among elderly women carrying *TNF-α* rs1800629 GG genotype. The adjusted ORs for *VDR* rs3782905 CG/CC genotype among elderly women carrying *TNF-α* rs1800629 AG/AA and GG genotypes were 0.1 (0.01, 0.98) and 3.54 (1.51, 8.30), respectively.

**Table 3:** Significant gene–gene interaction on overall and lumbar spine osteoporosis among women.

Gene 1	SNP 1	Gene 2	SNP 2	P for interaction^a^	
				Overall	LS
*TNF-α*	rs1800629	*VDR*	rs3782905	0.010	0.010

^a^: FDR p-value. LS: lumbar spine.

**Fig 2:**
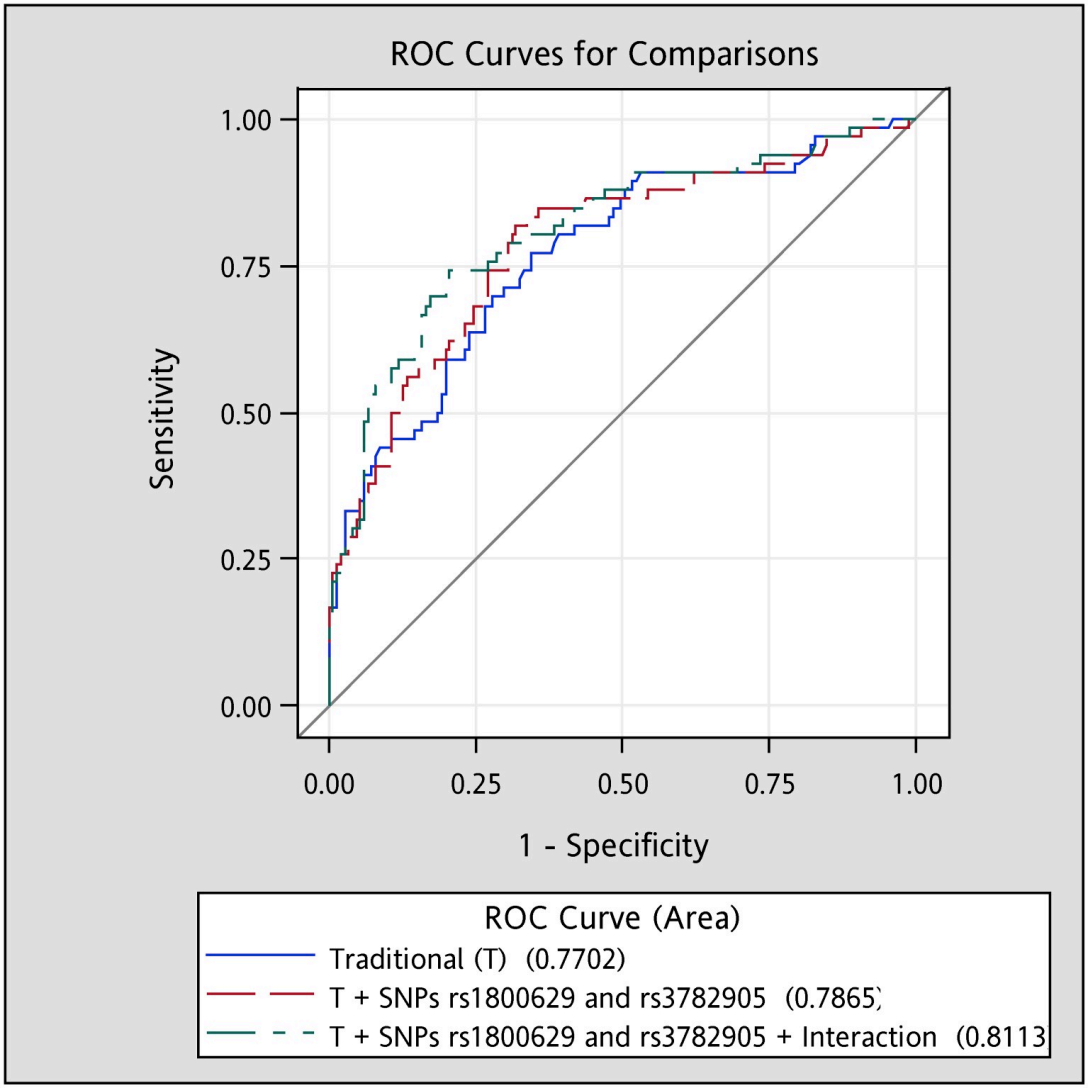
Areas under the receiver operating characteristics (AUROC) curve for overall OST among women. After taking additional age, BMI, physical activity, smoking, and drinking into account, the AUROC (95% confidence interval) for Traditional (T) model, “T + SNPs rs1800629 and rs3782905” model, and “T + SNPs rs1800629 and rs3782905 + Gene-gene interaction” model to predict overall osteroporsis were 0.77 (0.70, 0.84), 0.79 (0.72, 0.86), and 0.81 (0.75, 0.88), respectively. Significant differences were observed in the AUROC between Traditional and “T + SNPs rs1800629 and rs3782905 + Gene-gene interaction” models (p = 0.028). Interaction: interaction between *TNF-α* rs1800629 and *VDR* rs3782905.

**Fig 3:**
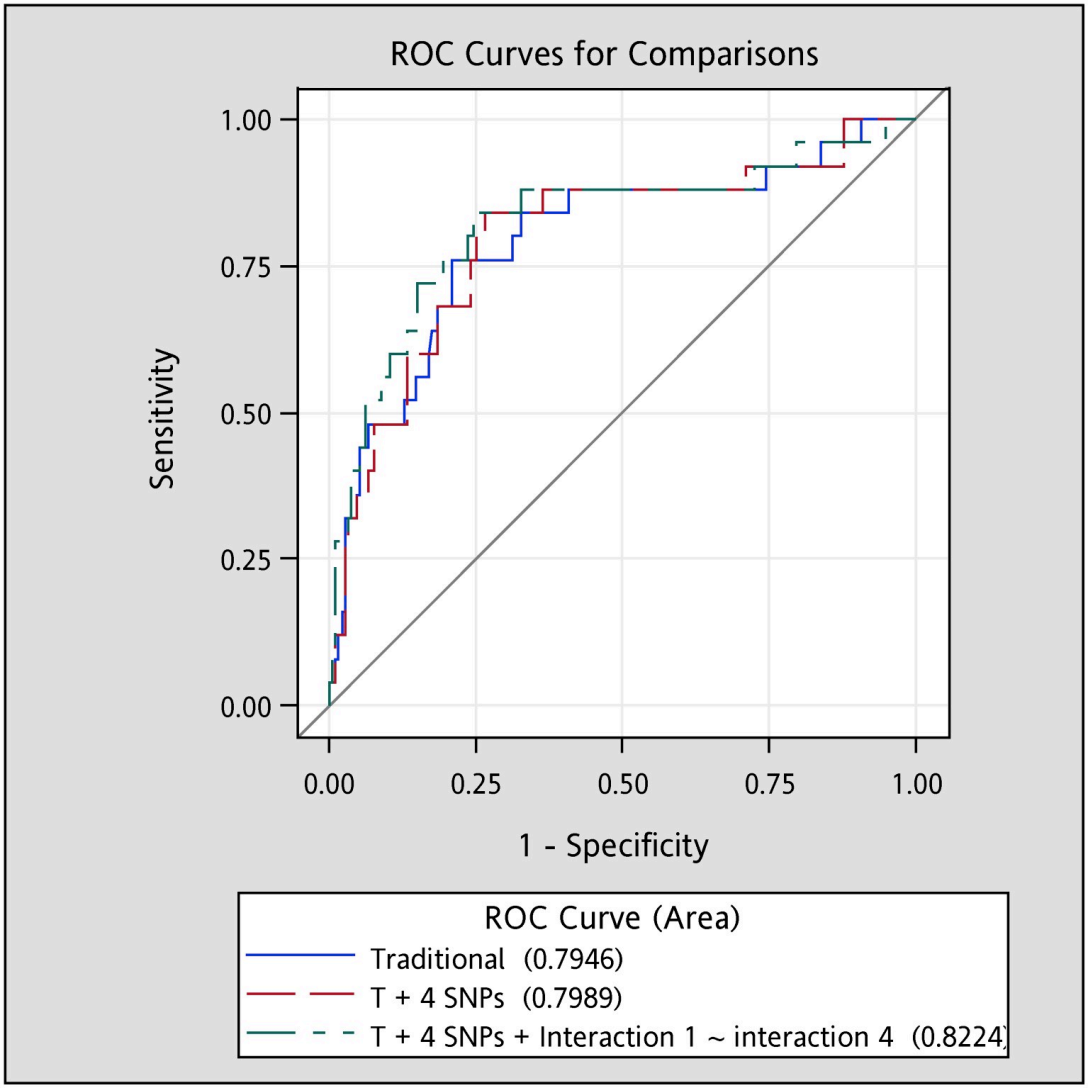
Areas under the receiver operating characteristics (AUROC) curve for lumbar osteoporosis among men. After taking additional age, BMI, physical activity, smoking, and drinking into account, the AUROC (95% confidence interval) for Traditional (T) model, “T + 4 SNPs” model, and “T + 4 SNPs + Physical activity-gene interactions 1–4” model to predict lumbar osteoporosis were 0.79 (0.69, 0.90), 0.80 (0.70, 0.90), and 0.82 (0.72, 0.93), respectively. No significant difference was observed in the AUROCs. These 4 SNPs included *VDR* SNPs rs7975232, rs1544410, rs2239185, and rs3782905. Interaction 1: interaction between physical activity and *VDR* rs7975232; Interaction 2: interaction between physical activity and *VDR* rs1544410; Interaction 3: interaction between physical activity and *VDR* rs2239185; and Interaction 4: interaction between physical activity and *VDR* rs3782905.

The AUROC curves of overall osteoporosis for traditional covariates, traditional covariates plus *TNF-α* rs1800629 and *VDR* rs3782905, and traditional covariates plus *TNF-α* rs1800629 and *VDR* rs3782905 and their interaction term were 0.77 (95% CI: 0.70, 0.84), 0.79 (0.72, 0.86), and 0.81 (0.75, 0.88), respectively (Fig 2). All values for AUROC curves showed good discrimination (> 0.7). In addition, significant difference was observed in AUROC values between the model with traditional covariates plus SNPs and their interaction term and that with traditional covariates only (P = 0.028). We did not detect significant interaction between SNPs in *TNF-α* and *VDR* genes with physical activity in elderly women and SNPs in *TNF-α* gene with physical activity in elderly men. Four significant interaction terms between SNPs in *VDR* gene (rs7975232, rs1544410, rs2239185, and rs3782905) with physical activity for osteoporosis at lumbar spine in men had been reported in our prior work [29]. Moreover, the AUROC curves for traditional covariates, traditional covariates plus four *VDR* SNPs, and traditional covariates plus four *VDR* SNPs and their interaction terms with physical activity were 0.79 (0.69, 0.90), 0.80 (0.70, 0.90), and 0.82 (0.72, 0.93), respectively (Fig 3). Similarly, all values for AUROC curves showed good discrimination (> 0.7). However, no significant difference was observed in AUROC values among the models with traditional covariates only, with traditional covariates plus SNPs, and with traditional covariates plus SNPs and their interaction term with physical activity (*p* > 0.05).

## Discussion

In this study, we examined the interaction between *VDR* gene and *TNF-α* gene on osteoporosis in Chinese elders in Taiwan to determine whether or not *VDR* gene is associated with the regulation of immune functions. The results showed that SNP in *TNF-α* rs1800629 interacted with rs3782905 in *VDR* gene on overall and lumbar spine osteoporosis among elderly women. Moreover, the model introducing these two SNPs along with their interaction between *TNF-α* and *VDR* genes had better prediction than that with traditional covariates on overall and lumbar spine among elderly women. The model considering traditional covariates along with interaction between physical activity with SNPs in *VDR* gene rs7975232, rs1544410, rs2239185, and rs3782905 had good discriminatory ability in predicting osteoporosis at lumbar spine. Our results could provide valuable information to further understand the genetic mechanisms underlying inflammation and vitamin D in osteoporosis among elderly women.

Many previous genetic studies explored the relationship of SNPs in *VDR* or *TNF-α* gene with osteoporosis [20, 28, 37–39]. However, no study simultaneously evaluated these two genes or examined their gene–gene or gene–physical activity interaction. A recent systematic review and meta-analysis explored the relationship between SNPs in *VDR* gene and osteoporosis risk in postmenopausal women. Their results showed that the association between *VDR* polymorphisms and osteoporosis risk in postmenopausal women varied among ethnic population groups. *VDR Bsml* and *Fokl* variants were associated with the increased risk of osteoporosis in Asian postmenopausal women but not in Caucasian postmenopausal women [28]. On the contrary, *VDR Apal* polymorphism was associated with the decreased risk of osteoporosis in Caucasian postmenopausal women [28] but not in Asian postmenopausal women. In the present study, the main or interactive effect of *VDR Bsml* (rs1544410) on osteoporosis was not observed, which was consistent with prior works [40–42]. On the contrary, we found that *VDR* rs3782905 polymorphism is interactive with *TNF-α* rs1800629 on overall and lumbar spine osteoporosis.

Aging is associated with chronic inflammation and is characterized by a systemic increase in plasma inflammatory mediators such as TNF-α [43]. TNF-α is an important inflammatory factor in the acute–phase response and plays various roles in the immune responses [44]. An imbalance in producing and releasing these cytokines has been linked with the appearance or worsening of age-related chronic conditions. A previous study reported that a high TNF-α level was observed in osteoporosis patients with maintenance hemodialysis of chronic renal failure for more than 33 months compared with those without osteoporosis [45]. Our prior study also reported that the haplotype lymphotoxin α gene, a member of the TNF superfamily, is associated with variations in TNF-α serum levels in community-dwelling elders [46]. Considering that genetic variations may influence cytokine production, we explored whether or not genetic variants modulating the inflammatory process may have the gene–gene effect or may interact with the effects of physical exercise on osteoporosis among elderly individuals. We especially focused on *TNF-α* and *VDR* genes because the elderly are often associated with hospitalization, severe infection, and disability [47, 48]. In addition, a possible strategy of preserving their functions on the immune system to cause less damage during aging could be established. Moreover, vitamin D is beneficial for the immune system and bone marrow [49]. A study correlated the vitamin D level with several profiles of the immune system, including CD4, CD8, and CD4/CD8 ratio among the elderly and found that vitamin D is mainly associated with CD8.

Age-related health conditions are complex phenotypes that are affected by interaction among many genes and environmental factors. However, no prior study investigated the interaction between inflammatory genes of *TNF-α* and *VDR* and the interactive effects of *TNF-α* gene with physical activity. In the current study, we found the interactive effect of SNP in *TNF-α* rs1800629 with rs3782905 in *VDR* gene on overall and lumbar spine osteoporosis among elderly women. This interactive effect may have occurred because the variants of *VDR* gene regulating the vitamin D receptor allow our body to respond to vitamin D. This process may protect against the inflammation induced by the level of the *TNF-α* variants. Thus, elders with variants of TNF-α and *VDR* genes that are associated with low vitamin D receptor may need food supplements with vitamin D.

Our study has two limitations. First, the small sample size for examining gene–gene and gene–physical activity interactions was relatively small. Therefore, we may have omitted important variants associated with osteoporosis because of the need for stratification analysis for gender that limited the statistical power. Second, we only genotyped few candidate variants reported in literature. Our observed effect may indicate the true susceptibility allele or effect due to LD to the “true” susceptibility allele. Sequencing of *TNF-α* and *VDR* genes may be needed in the future study.

Our study also had strengths. First, our study was the first to assess the potential gene–gene interactions between the three SNPs in the *TNF-α* gene and four SNPs in the *VDR* gene and osteoporosis and the interactions among the three SNPs in the *TNF-α* gene with the physical activity among Chinese elders dwelling in community. Second, our study was a community-based representative sample of Chinese elderly population. Hence, the likelihood of selection bias is minimized. Therefore, our results can be generalized to other Chinese elderly populations. Third, our laboratory had set up the standardized procedure for stringent quality control on the measurement of genotypes.

## Conclusion

Our data suggest that the interaction of the CG/CC genotype of *VDR* rs3782905 with *TNF-α* rs1800629 GG genotype is associated with increased risks of overall and lumbar spine osteoporosis among elderly women. Moreover, the main and interactive effects of these variants significantly improve the prediction ability on overall osteoporosis among elderly women. These findings help depict the genomic mechanisms and provide new insights into genetic screen testing on osteoporosis. Education programs focusing on food supplement for vitamin D should target the elderly who are genetically predisposed for osteoporosis prevention.
